# Accuracy of frozen section for HPV-Associated squamous cell carcinoma of unknown primary

**DOI:** 10.1016/j.oraloncology.2026.107861

**Published:** 2026-01-19

**Authors:** Sindhura Sridhar, Annie Moroco, Shravan Gowrishankar, Mitra Mehrad, Kim Ely, James S. Lewis, Madalina Tuluc, Stacey Gargano, Melanie Hicks, Kyle Mannion, Arielle G. Thal, Adam J. Luginbuhl, Joseph M. Curry, David M. Cognetti, Michael C. Topf

**Affiliations:** a Vanderbilt University Medical Center, Department of Otolaryngology – Head and Neck Surgery, 1215 21^st^ Ave. S., Nashville, TN 37232, United States; b Thomas Jefferson University, Department of Otolaryngology – Head and Neck Surgery, 111 S. 11^th^ St., Philadelphia, PA 19107, United States; c Vanderbilt University Medical Center, Department of Pathology, Microbiology, and Immunology, 1211 Medical Center Drive, Nashville, TN 37232, United States; d Mayo Clinic Arizona, Department of Laboratory Medicine and Pathology, 5777 E Mayo Blvd, Phoenix, AZ 85054, United States; e Thomas Jefferson University, Department of Pathology and Genomic Medicine, 111 S. 11^th^ St., Philadelphia, PA 19107, United States

## Abstract

**Introduction:**

Current guidelines for the management of metastatic squamous cell carcinoma of unknown primary (SCCUP) recommend submission of suspicious primary sites for frozen section analysis (FSA). This study aims to investigate the diagnostic accuracy of FSA for identification of HPV-associated SCCUP.

**Methods:**

A retrospective cohort study of patients with biopsy-proven p16-positive SCCUP who underwent diagnostic operation at two tertiary care institutions was performed. Sensitivity, specificity, PPV, and NPV of diagnostic FSA were assessed.

**Results:**

77 patients were included in analysis. 66 patients underwent definitive TORS (diagnostic TORS operation with subsequent neck dissection after identification of the occult primary tumor), 7 patients underwent diagnostic TORS (TORS to identify occult primary tumor, no neck dissection), and 4 patients underwent direct laryngoscopy and biopsy only. Primary tumors were identified in 63 patients (82%) with a mean tumor size of 1.1 cm. There was no significant difference in size between patients whose tumor was identified on FSA (mean 1.1 cm) and on permanent only (mean 0.9 cm) (p = 0.26). The sensitivity, specificity, PPV, and NPV of FSA for SCCUP was 86%, 100%, 100%, and 86%, respectively. Diagnostic frozen specimens included 52 direct laryngoscopy biopsies and 69 TORS excisions. In the biopsies, sensitivity was 100% and NPV was 100%, whereas in the TORS-excised specimens, sensitivity was 77% and NPV was 77%.

**Conclusions:**

In this case series of 77 patients with SCCUP, the sensitivity and NPV of FSA for identification of the primary tumor was over 85%. FSA is valuable during diagnostic operation for SCCUP.

## Introduction

Squamous cell carcinoma of unknown primary (SCCUP) of the head and neck is characterized by cervical nodal metastasis with an occult primary tumor, most commonly associated with high-risk human papillomavirus (HPV).[1,2] Given the association of SCCUP with HPV, occult, unknown primary tumors are most often located in the oropharynx and specifically the tonsillar tissue containing palatine tonsils and base of tongue (lingual tonsil).^3^ Initial diagnostic workup for head and neck SCCUP includes a thorough history, physical examination including fiberoptic laryngoscopy, cervical nodal biopsy with either fine needle aspiration (FNA) or core needle biopsy (CNB), and imaging including computed tomography (CT) and positive emission tomography (PET).[3–5].

If localization of the primary tumor on physical examination and imaging is unsuccessful, diagnostic or therapeutic surgery is indicated. [6] Current recommendations by the American Society of Clinical Oncology (ASCO) for the management of HPV-associated SCCUP include submission of tissue specimens from suspicious primary sites including biopsies, palatine tonsillectomies, and lingual tonsillectomies for frozen section analysis (FSA).[6] Reported rates of intraoperative primary tumor identification range from 20 to 30% in direct laryngoscopies with biopsy (DLB) and 72.3–82.7% using transoral robotic surgery (TORS). However, a recent study found the sensitivity of FSA for intraoperative identification of HPV-associated SCCUP to be less than 50%, calling into question the utility of FSA.[7] Given the critical role of FSA in the identification and diagnosis of HPV-associated SCCUP in current practice, it is imperative that its diagnostic accuracy is thoroughly evaluated and understood.

In this two-institution retrospective review, we further investigated the diagnostic accuracy of FSA in the HPV-associated SCCUP patient population. The primary aim of this study was to evaluate the sensitivity, specificity, positive predictive value (PPV), and negative predictive value (NPV) of FSA for intraoperative identification of HPV-associated SCCUP. In addition, we aimed to assess whether certain clinical and surgical factors impact the accuracy of FSA in this setting.

## Methods

### Study population

This study was approved by the Vanderbilt University Medical Center (IRB #241989) and the Thomas Jefferson University Institutional Review Boards (IRB #14D.448). Informed consent was not required by the IRB due to the use of deidentified retrospective data. This retrospective analysis used a multi-institutional cohort of patients from two participating academic institutions in the United States. The names and data from each institution were deidentified to Institution 1 and Institution 2 prior to combined analysis. The Strengthening the Reporting of Observational Studies in Epidemiology (STROBE) reporting guidelines were followed.

We retrospectively reviewed patients with biopsy-proven p16-positive SCCUP who underwent DLB and/or TORS for identification of the primary tumor between May 2012 and September 2024. Patients were considered to have SCCUP if they presented with a neck mass with no definitive evidence of primary tumor on clinical examination (complete head and neck physical examination and flexible laryngoscopy) or radiographic examination (CT with contrast, magnetic resonance imaging [MRI], PET). All patients underwent cervical nodal biopsy with FNA, CNB, or excisional biopsy with p16 immunostaining to verify disease etiology. Patients with prior head and neck cancer and p16-negative or p16-indeterminate SCCUP were excluded. Patients who did not have intraoperative FSA were also excluded.

### Data Collection

Demographic data were collected for each patient, including age at the time of surgery, sex, race, ethnicity, smoking status, pack-years smoked, alcohol use, and history of tonsillectomy. Since the primary objective of the study was to assess the utility of FSA for identification of the unknown primary, operative details and pathological data were recorded, including procedure(s) performed and intraoperative specimens submitted for FSA. Diagnostic procedures were categorized as DLB only, diagnostic TORS (defined as TORS palatine or lingual tonsillectomy for primary tumor localization without neck dissection), and definitive TORS (defined as a diagnostic and definitive TORS operation with subsequent ipsilateral or bilateral neck dissection after identification of the occult primary tumor).

For each intraoperative specimen submitted for FSA, the location, type of specimen (TORS excision vs. direct laryngoscopy biopsy), FSA findings, and permanent section findings were collected, as well as the subspecialty status of the reporting pathologist. Only intraoperative specimens submitted for diagnostic purposes (identification of the occult primary tumor), were included in this study. Once the primary site was localized, subsequent intraoperative specimens submitted for FSA margins after identification of the primary tumor were excluded.

In addition, tumor data were obtained, including American Joint Committee on Cancer (AJCC) 8th edition clinical T and N stage, pathological T and N stage, and whether the tumor was identified intraoperatively (yes/no). If the tumor was identified, oropharynx subsite, tumor laterality, size of the tumor on permanent examination, and presence or absence of perineural invasion (PNI) and/or lymphovascular invasion (LVI) were recorded.

### Statistical analysis

All statistical analysis was performed using R (R version 4.4.1, https://www.r-project.org). The utility of FSA was evaluated by calculating sensitivity, specificity, predictive value (PPV), and negative predictive value (NPV), using the results of permanent section analysis as the gold standard. The results were further analyzed by procedure type (DLB vs. TORS excision). Associations of clinicopathologic characteristics with likelihood of intraoperative primary tumor identification were assessed using the Mann-Whitney *U* test for continuous variables and chi-square test for categorical variables, and subsequently with logistic regression.

### Intraoperative approach to the occult primary tumor

The intraoperative approach at Institution 1 and Institution 2 is similar. The diagnostic operation begins with palpation of the oropharynx and direct laryngoscopy with use of operative telescope. If any obvious lesions are identified, a biopsy is performed using cupped forceps through the laryngoscope and is sent for FSA. If no lesion is seen or biopsy is negative, then the DaVinci robotic surgical system (Intuitive Surgical, Inc., California, USA) is brought into the field and an endoscopic exam of the oropharynx is performed. If a primary site continues to not be appreciated, a directed robotic simple resection of the ipsilateral palatine tonsil and/or ipsilateral lingual tonsil is performed in a stepwise fashion. The diagnostic operation may be modified based on clinical history and radiographic findings. For example, if the patient had a history of prior tonsillectomy, the patient may receive a contralateral lingual tonsillectomy if the ipsilateral lingual tonsillectomy is negative on frozen section. If the patient has bilateral nodes on imaging, a bilateral lingual tonsillectomy may be performed. It is the practice of both institutions to submit all suspicious diagnostic specimens for FSA and not defer to permanent section analysis for primary tumor identification.

Submitted specimens are oriented with the surgeon who may or may not indicate an area of concern. If no areas of concern are indicated, the specimen is inked, serially sectioned, and entirely submitted for FSA. If an area of concern is indicated, it is submitted for FSA and margins are assessed; if no primary is identified, the remainder of the specimen is serially sectioned (3–4 mm per section) and entirely submitted. Individual sections are frozen with Optimal Cutting Temperature (OCT) embedding medium and cut at 5 μm with a cryostat.

At Institution #1, two different levels (slides) per section are assessed for every specimen; an additional level may be assessed if no tumor is seen on the initial slides. A head and neck subspecialty trained pathologist is available for consultation but is not always the primary pathologist evaluating each frozen section. At Institution #2, three different levels per section are assessed for all head and neck specimens and selected others (bile duct margins in pancreaticobiliary cancer), as an attempt to minimize false negative frozen sections. All specimens are evaluated by a head and neck subspecialty trained pathologist.

## Results

### Patient Characteristics

The study cohort included 77 patients with p16-positive SCCUP with a mean age of 60.5 years (range 41–86). There were 74 males (96%), 70 (91%) of whom were white. Forty-four patients (57%) were current or former smokers. Eleven patients (14%) had undergone prior tonsillectomy. Most patients were AJCC 8th edition cN1 (70 patients [91%]).

Sixty-six patients (86%) underwent definitive TORS and concurrent neck dissection including 6 patients that underwent definitive resection as a separate procedure after identification of the initial primary tumor. Seven patients (9%) underwent diagnostic TORS, 3 patients (4%) underwent DLB alone, and 1 patient (1%) underwent DLB followed by definitive non-robotic resection.

Among the patients who underwent definitive TORS and neck dissection, 33 patients received no adjuvant treatment, 21 patients received adjuvant radiation therapy, and 12 patients received adjuvant chemoradiation. Nine patients underwent definitive chemoradiation and two patients underwent definitive radiation therapy. Complete patient demographic and clinical data are shown in Table 1.

### Intraoperative identification of the primary tumor

Primary tumors were identified in 63 patients (82%), including 49 (64%) that were identified intraoperatively. Fourteen tumors were identified on permanent section only. Of these, eight patients had false negatives (frozen section specimen negative for carcinoma with final read of corresponding section positive for p16 + squamous cell carcinoma) and six were identified on specimens that were not submitted for FSA. Fourteen patients (18%) did not have primary tumors identified after FSA and permanent section analysis of submitted tissue specimens. There was no significant difference in size between patients whose tumor was identified intraoperatively (mean 1.1 cm) and on permanent sections only (mean 0.9 cm) (p = 0.26).

There were 121 total specimens submitted for FSA, including 52 biopsies via direct laryngoscopy and 69 TORS excisions (ipsilateral palatine or lingual tonsillectomy). Primary tumors were identified intraoperatively in 25 biopsy specimens (48%) and 30 TORS excisions (43%). The mean number of frozen sections per patient was 1.6 (median 1, range 1–4), with the primary tumor most often identified on the final frozen section submitted.

Of the primary tumors identified, 27 (43%) were found in the ipsilateral tonsil, 25 (40%) in the ipsilateral tongue base, 6 (10%) in the ipsilateral glossotonsillar sulcus, 1 (2%) in the central tongue base, and 4 (6%) in multiple anatomic subsites. There were no isolated contralateral tumors. Primary tumor sizes ranged from 0.2 to 2.5 cm, with mean and median sizes of 1.1 cm and 1.0 cm, respectively.

The sensitivity, specificity, PPV, and NPV of FSA for SCCUP was 86% (95% CI 75%–93%), 100% (95% CI 94%–100%), 100% (95% CI 94%–100%), and 86% (95% CI 76%–94%), respectively, resulting in an overall accuracy of 93% (95% CI 87%–97%) (Table 2). In the biopsy specimens obtained during direct laryngoscopy, sensitivity was 100% (95% CI 86%–100%) and NPV was 100% (95% CI 87%–100%), with an accuracy of 100% (95% CI 93%–100%), whereas in the TORS excisions to locate the occult primary, sensitivity was 77% (95% CI 61%–89%) and NPV was 77% (95% CI 61%–89%), with an accuracy of 87% (95% CI 77%–94%). There was also no significant difference in sensitivity by institution (Institution 1 (81%) vs. Institution 2 (88%), p = 0.20).

Primary tumors from patients who were current or former smokers were equally likely to be identified compared to patients who were never smokers (36/44 [82%] vs 27/33 [82%], p = 1.00) (Table 3). The primary was identified in 8 of the 11 patients (73%) with a history of a tonsillectomy, with 7 of the 8 primary tumors identified intraoperatively on FSA. There was no significant difference in age, sex, race, tumor subsite, or history of tonsillectomy between patients whose tumor was identified intraoperatively and on permanent sections only.

False negatives occurred in 9 frozen specimens in 8 total patients (Table 4). All false negatives occurred in TORS-excised, rather than biopsy specimens. Sampling error (tumor not present on the levels examined via FSA but present in the remaining specimen examined during permanent section analysis) was the most common cause of false negatives and was noted in 7 specimens (6% overall rate of sampling error).[8] There was no significant difference between rate of sampling error at both institutions when looking at all frozen sections (Institution 1 (9%) vs Institution 2 (4%), p = 0.52) or when analyzing only the cases where the primary tumor was identified (on either frozen or permanent section analysis) (Institution 1 (11%) vs Institution 2 (5%), p = 0.52). The other cause of false negatives was interpretation error (tumor is present on the frozen section slides and was not recognized by the pathologist), occurring in 2 specimens (2% overall rate of interpretation error).[8] One of these cases was signed out by a subspecialty trained head and neck surgical pathologist and one case was signed out by a non-head and neck surgical pathologist. There were no false positives (tumor diagnosed on FSA but ultimately determined to not represent actual SCC).

## Discussion

In this multi-institutional retrospective study of 77 patients with HPV-associated SCCUP, the sensitivity, specificity, PPV, and NPV of FSA for intraoperative identification of the primary tumor was 86%, 100%, 100%, and 86%, respectively, resulting in an overall diagnostic accuracy of 93%. The accuracy of FSA was higher for biopsy specimens obtained via direct laryngoscopy at 100% compared to specimens excised to locate the unknown primary tumor via TORS at 87%. There was no difference in age, sex, race, tumor subsite, tumor size, or history of tonsillectomy between patients whose tumor was identified intraoperatively and those who were not. FSA diagnostic errors were all false negatives and were most often caused by sampling error. False negatives only occurred in TORS-excised specimens.

FSA is a routine aspect of head and neck oncologic surgery and has shown high accuracy in identifying tumors and determining head and neck surgical margins across cancer subsites including the oropharynx. [9–12] Although FSA is used routinely for intraoperative identification of the unknown primary, there is limited literature evaluating the accuracy of FSA for this purpose. A recent single institution, retrospective study by Awad et al. investigating FSA for intraoperative identification of HPV-associated SCCUP of 47 patients showed a sensitivity of 49%, NPV of 34%, and diagnostic accuracy of 60%, values much lower than the findings of our analysis.[7] In addition, Awad et. al found a significant difference in the size of tumors found intraoperatively in comparison to those identified in permanent section analysis only, and found no significant difference in tumor subsite, margin status, tumor laterality, tobacco use, history of tonsillectomy, and specimen type between tumors identified intraoperatively and tumors identified on permanent section analysis. All intraoperative consultations in the Awad study were performed by subspecialty-trained head and neck pathologists. The notable differences in sensitivity, NPV, and diagnostic accuracy between the findings of Awad et al. and the current study underscore the difficulties of FSA particularly for disease processes such as HPV-associated SCCUP which pose a challenge to identify and diagnose.

There are numerous factors that may contribute to the variability of FSA for SCCUP. Errors in FSA can be caused by either sampling error or interpretive error.[8,9,13–16] Intraoperatively, FSA outcomes may be impacted by small amounts of tumor present histologically, specimen distortion due to crush or cautery artifact from TORS, artifact from freezing, and the type of specimen submitted (biopsy vs. complete tumor excision), all of which can affect tumor on the histologic slides and contribute to sampling and interpretive errors.[8,9,13,14,16] During histopathological processing, distortion of tissue architecture due to the freezing process, uneven sectioning, or poor staining can predispose to interpretive error, and inadequate or superficial sectioning can also contribute to sampling error.[8,9,13,14,16] We did not find any difference in likelihood of identifying the occult primary based on specimen size, specimen type, or tumor subsite. However, biopsy specimens had greater sensitivity and fewer false negatives compared to TORS excisions (lingual/palatine tonsillectomy or oropharyngectomy). This is not surprising as biopsies are typically directed at visible lesions and are smaller in size compared to TORS-excised specimens. Therefore, the diagnostic yield is likely to be higher and there is less chance of missing a small primary tumor that may be deeper within the specimen.

We found that the majority of frozen section errors in our study cohort were caused by sampling error, consistent with prior studies. [9,16] Notably, the institutions represented in this study have different approaches to histopathological processing of unknown primary specimens. Although the rate of sampling error was not significantly different between both institutions, there was a greater rate of error in Institution 1 (9%), which primarily uses two-level sectioning, compared to Institution 2 (4%), which primarily uses three-level sectioning, raising the question as to whether additional levels should be routinely assessed for HPV-associated SCCUP. There are few prior studies investigating how sectioning affects sampling error. Olson et al. showed that cutting a third histological level can reduce frozen section sampling error in head and neck cancer specimens.[8] In contrast, Shan et al showed that re-evaluation of slides by a head and neck subspecialty trained pathologist, but not additional levels or p16 staining, improved identification of HPV-associated primary tumors when they were not initially identified on frozen or permanent section analysis.[17] In an occult unknown primary tumor that is often small and difficult to identify, an additional level may improve diagnostic yield, however, further investigation is needed to more fully evaluate the value of this practice for HPV-associated SCCUP.

**Fig. 1.**
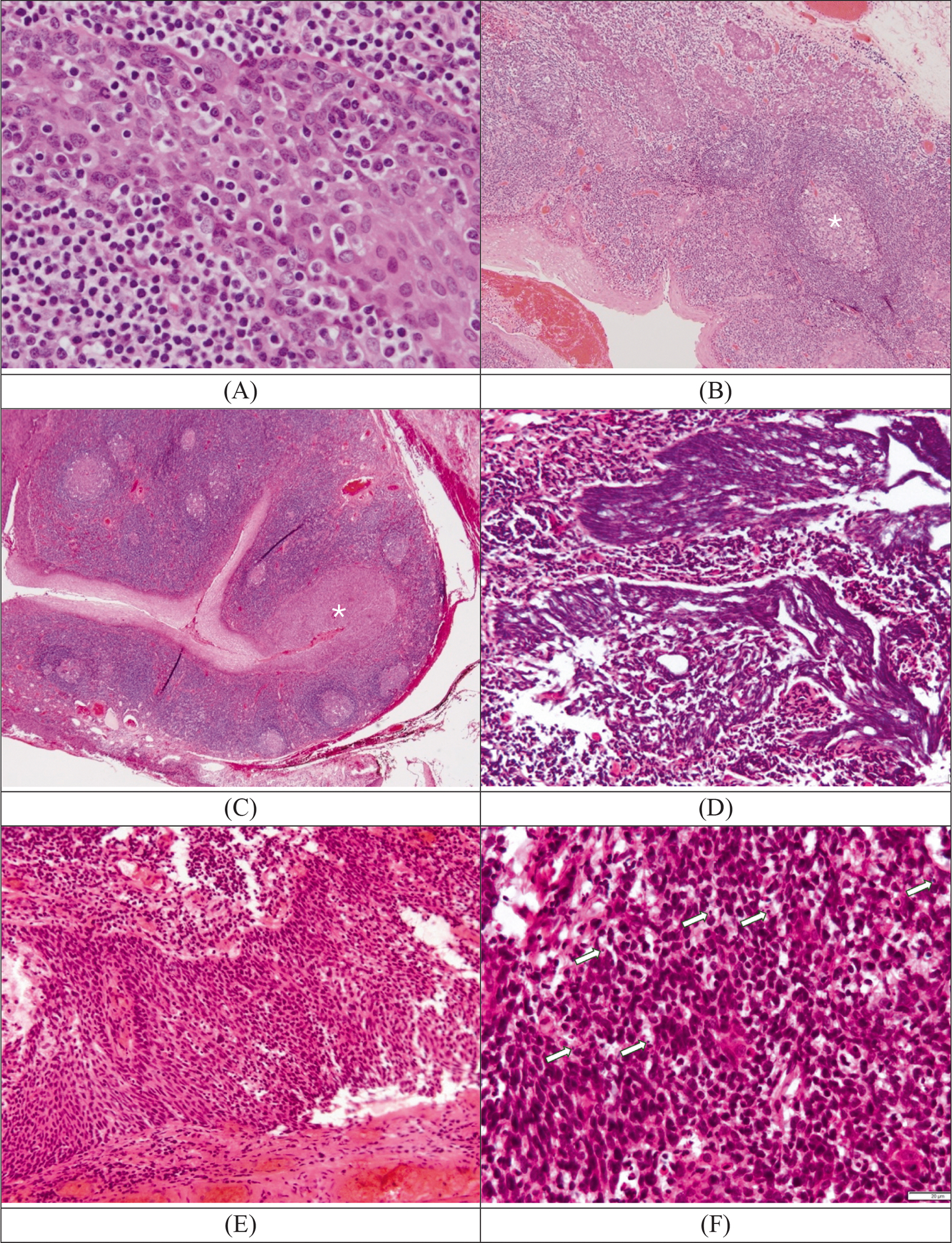


Small foci of HPV-associated oropharyngeal squamous cell carcinoma (OPSCC) may be difficult to distinguish from non-neoplastic tonsillar tissue on FSA (Figs. 1A). As the appearance of HPV-associated OPSCC is typically non-keratinizing or “basaloid”, it may blend into the background lymphoid stroma (Figs. 1B and 1C). Unlike conventional squamous cell carcinomas, it often does not elicit a desmoplastic response. Furthermore, normal or “expected” tonsillar architecture can be distorted by frozen and thermal artifact and mimic a carcinoma (Fig. 1D). Helpful histopathologic features that can assist in its recognition include a tendency for the tumor to “cleave” or separate from surrounding tissue (Fig. 1E). Finally, HPV-associated OPSCCs often contain apoptotic bodies (Fig. 1F) or overt necrosis, as well as atypical hyperchromatic cells which will be absent in normal tonsillar crypts.

The results of this study emphasize the importance of close communication between head and neck surgical oncologists and pathologists as well as the availability of subspecialty-trained head and neck pathologists for effective management of SCCUP. Given the rising incidence of HPV-associated OPSCC, it is expected that the rate of HPV-associated SCCUP will continue to increase.[3] Although innovative imaging modalities such as transoral ultrasound, transcervical ultrasound, and narrow band imaging have augmented evaluation of these patients, directed biopsies and TORS continue to be a mainstay of diagnosis when the primary tumor cannot be located on physical exam or imaging.[3–5] As such, FSA continues to be a valuable adjunct to the work-up of SCCUP.

### Limitations

This study has several limitations. Our study cohort includes patients with HPV-associated SCCUP from two different academic institutions and is comprised largely older White, male patients. Although cohorts from multiple institutions were combined, the study size remains small, which limits the generalizability of our results. In addition, there are slight differences in protocols between institutions for diagnosis and workup of SCCUP which we were not able to account for in our analysis. At Institution #2, all specimens are evaluated by a head and neck subspecialty trained pathologists, whereas at Institution #1, a head and neck subspecialty trained pathologist is available for consultation but is not always the primary pathologist evaluating each frozen section. The rates of DLB, diagnostic TORS, and definitive TORS also vary at each institution. Despite these differences, the accuracy of FSA remains high and did not significantly change between institutions. Another limitation is that the efficacy of FSA in identifying very small (<0.5 cm) primary tumors may be under-evaluated in this study, as tumors that remained unidentified after diagnostic operation are more likely to be too small to detect on clinical examination or imaging. Our cohort (median 1.1 cm, range 0.2–2.5 cm) is comparable to sizes of identified clinically occult primary tumors in published literature (median size 0.8–1.3 cm, range 0.3–3.8 cm).[18–21] Therefore, we believe that this study is representative of the utility of FSA with typical presentation of HPV-associated SCCUP. Future retrospective studies should aim to include a variety of institutions and regions to generate a larger cohort size and more comprehensively evaluate the utility of FSA for SCCUP; this research question would also benefit from prospective study design to control for institutional differences.

### Conclusion

In this case series of 77 patients with SCCUP, the sensitivity and NPV of frozen section analysis for identification of the primary tumor was over 85%. Consistent with ASCO recommendations, FSA is reliable in this population, regardless of tumor size, tumor subsite, type of operation, and specimen type. FSA is a valuable adjunct tool to diagnostic and definitive operation for SCCUP.

## Figures and Tables

**Table 1:** Characteristics of Patients with HPV-associated Squamous Cell Carcinoma of Unknown Primary.

	Number of Cases	%

**Age**		
Mean	60.5	
Range	41–86	
**Gender**		
Male	74	96
Female	3	4
**Self-Reported Race/Ethnicity**		
Caucasian	70	91
African-American	2	3
Hispanic	2	3
Multiracial	2	3
Asian	1	1
**Tobacco Use—Current or Former**		
Yes	44	57
No	33	43
**Tonsillectomy as a Child**		
Yes	11	14
No	66	86
**Procedure**		
Definitive TORS	65	84
Diagnostic TORS	7	9
Diagnostic Laryngoscopy With Biopsy Only	5	6
**Specimens Submitted**		
Biopsy	52	43
Complete Tumor Resection	69	57
**Pathologic T Stage**		
T0	14	18
T1	54	70
T2	5	6
TX	4	5
**Pathologic N Stage**		
N1	68	88
N2	2	3
NX	7	9

**Table 2:** Diagnostic Accuracy of Intraoperative Frozen Sections for HPV-associated SCCUP.

	Sensitivity	Specificity	PPV	NPV	Accuracy

Overall	0.86	1.00	1.00	0.86	0.93
Biopsy Specimens	1.00	1.00	1.00	1.00	1.00
Excisions	0.77	1.00	1.00	0.77	0.87

**Table 3:** Characteristics of Human Papillomavirus-Associated Squamous Cell Carcinoma of Unknown Primary Identified Intraoperatively.

	No. (%) (Total = 49)	Univariate Analysis (OR [95% CI])	Multivariate Analysis (OR [95% CI])

**Age (Mean [±SD])**	59.4 (9.4)	1.00 (0.99–1.01)	—
**Gender**			
Female	0	1 [ref]	—
Male	49 (100)	2.20 (0.98–4.96)	
**Self-Reported Race/Ethnicity**			
Other	5 (10)	1 [ref]	—
Caucasian	44 (90)	0.94 (0.66–1.34)	
**Tobacco Use—Current or Former**			
No	19 (39)	1 [ref]	1 [ref]
Yes	30 (61)	1.14 (0.92–1.40)	1.15 (0.92–1.43)
**Tonsillectomy as a Child**			
No	42 (86)	1 [ref]	—
Yes	7 (14)	1.12 (0.82–1.53)	
**Specimens Submitted**			
Biopsy	38 (51)	1 [ref]	1 [ref]
Excision	37 (49)	0.93 (0.72–1.22)	0.92 (0.70–1.31)
**Tumor Size (Mean [±SD])**	1.1 (0.6)	1.12 (0.93–1.36)	1.09 (0.88–1.34)
**Tumor Subsite**			
Base of Tongue	21 (43)	1 [ref]	1 [ref]
Tonsil	23 (47)	1.05 (0.84–1.30)	1.02 (0.80–1.29)
Other	5 (10)	**0.74 (0.55–0.99)**	0.92 (0.57–1.10)

**Table 4:** Characteristics of False Negative Intraoperative Frozen Section Results.

	Negative on Frozen/Positive on Final (N = 8) [No. (%)]

**Age, mean (±SD)**	60.5 (±8.3)
**Gender**	
Female	1 (14)
Male	7 (86)
**Self-Reported Race/Ethnicity**	
Caucasian	8 (100)
Other	0 (0)
**Tobacco Use—Current or Former**	
No	6 (75)
Yes	2 (25)
**Tonsillectomy as a Child**	
No	7 (88)
Yes	1 (11)
**Procedure**	
Diagnostic Laryngoscopy with Biopsy Only	0 (0)
Diagnostic TORS	2 (25)
Definitive TORS	6 (75)
**Specimens Submitted**	
Biopsy	0 (0)
Excision	9 (100)
**Tumor Size, cm (±SD)**	0.7 (0.3)
**Tumor Subsite**	
Base of Tongue	2 (25)
Tonsil	3 (38)
Other	3 (38)
**Type of Error**	
Sampling	7 (78)
Interpretive	2 (22)
